# Correction to “Rett Syndrome: The Natural History Study Journey”

**DOI:** 10.1002/cns3.70004

**Published:** 2025-04-18

Percy AK, Benke TA, Marsh ED, Neul JL. Rett syndrome: The Natural History Study Journey. *Ann Child Neurol Soc*. 2024;2(3):189–205. doi: 10.1002/cns3.20086.

**Table 1:** [Table 1 failed to include the site “Gillette Children's Hospital.”].

Enrollment sites	Investigators
Children's Hospital of Philadelphia/University of Pennsylvania	Eric Marsh, MD, PhD
University of Colorado‐Denver	Tim Benke, MD, PhD
Cincinnati Children's Hospital	Shannon Standridge, DO
Vanderbilt Medical Center	Jeffrey L Neul, MD, PhD, Sarika Peters, PhD, and Cary Fu, MD
Rush Memorial Medical Center	Peter Heydemann, MD
Washington University (St. Louis)	Robin Ryther, MD, PhD; Judith Weisenberg, MD
Children's Hospital‐Oakland/UCSF	Mary Jones, MD†
University of California San Diego	Jeffrey L Neul, MD, PhD and Richard Haas MD
University of Rochester^1^	Alex Paciorkowski, MD
Cleveland Clinic ‐ CDD only	Elia Pestana‐Knight, MD
Gillette Children's Hospital	Art Beisang, MD and Timothy Feyma, MD
Baylor College of Medicine^2^	Daniel Glaze, MD and Bernhard Suter, MD, PhD
Greenwood Genetic Center^2^	Steven Skinner, MD
Boston Children's Hospital^2^	Walter Kaufmann, MD, David Lieberman, MD, PhD, and Heather Olson, MD
University of Alabama at Birmingham^2^	Alan Percy, MD and Amitha Ananth, MD

We apologize for this error.

